# Fusion of Personalized Federated Learning (PFL) with Differential Privacy (DP) Learning for Diagnosis of Arrhythmia Disease

**DOI:** 10.1371/journal.pone.0327108

**Published:** 2025-07-11

**Authors:** Syed Mohsin Bokhari, Sarmad Sohaib, Muhammad Shafi

**Affiliations:** 1 Department of Electrical and Computer Engineering, University of Engineering and Technology, Taxila, Pakistan; 2 Department of Electrical and Electronic Engineering, University of Jeddah, Jeddah, Saudi Arabia; 3 School of Computing, Ulster University, Belfast, United Kingdom; Polytechnic University of Marche: Universita Politecnica delle Marche, ITALY

## Abstract

This paper presents a novel privacy-preserving architecture, a fusion of Federated Learning with Personalized Models and Differential Privacy (FLPMDP), for diagnosing arrhythmia from 12-lead electrocardiogram (ECG) signals. The architecture supports collaborative training in decentralized healthcare institutions without exposing sensitive patient information. By employing gated recurrent units (GRUs) for temporal sequence modeling along with feature fusion techniques and local differential privacy enforcement, FLPMDP ensures robust classification performance with data confidentiality. The architecture is evaluated on four experimental setups and demonstrates significant performance gain over centralized and federated baseline models. An empirical experiment on a large ECG dataset of 10,646 recordings indicates that the FLPMDP approach achieves an average accuracy of 93.71%. The FLPMDP approach yields F1-scores of 0.98, 0.93, 0.88, and 0.89 for sinus bradycardia (SB), atrial fibrillation (AFIB), supraventricular tachycardia (GSVT), and sinus rhythm (SR), respectively. Additionally, FLPMDP recorded a specificity up to 0.98, with a Kappa score of 0.8971 and a Matthews Correlation Coefficient of 0.9042, indicating high diagnostic accuracy and model strength. Comparative analysis against state-of-the-art methods—such as CNN, ResNet, and attention-based RNNs—indicate that FLPMDP consistently outperforms current models in accuracy, sensitivity, and robustness when facing non-IID data conditions. In the context of this research, federated learning is highly pertinent to modern healthcare, enabling secure and collaborative model training across institutions while complying with data privacy. The proposed FLPMDP framework offers a scalable and privacy-compliant solution for real-time arrhythmia detection, marking a step forward in deploying trustworthy artificial intelligence for decentralized medical diagnostics.

## 1 Introduction

**Fig 1:**
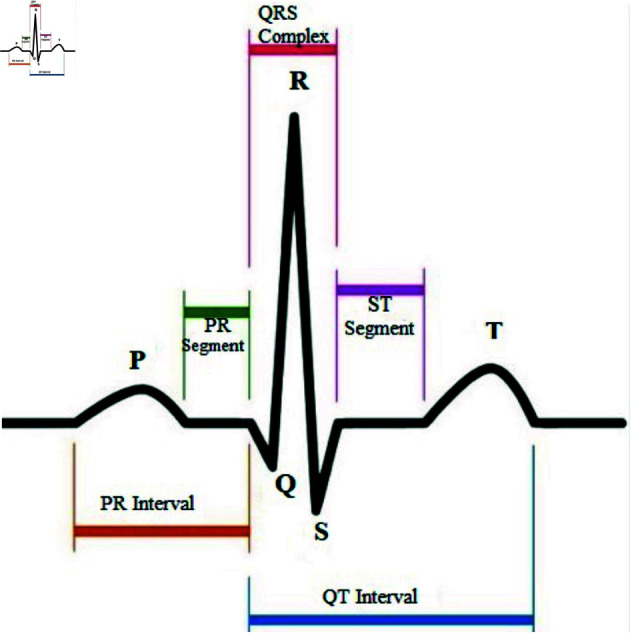
The structure of a typical ECG signal.

According to statistics from the World Health Organization (WHO), heart disease is the leading cause of death among chronic diseases. Each year, nearly 17.7 million people succumb to cardiovascular diseases (CVD), representing 31% of all global deaths [1]. The electrocardiogram (ECG) represents the electrical activity produced by the heart during heartbeats. ECG is widely used for diagnosing various heart diseases, including arrhythmia, myocardial infarction, atrial fibrillation, coronary artery disease, and heart block. Typically, an ECG is obtained by electrodes affixed to the patient’s skin, which capture the electrical alterations occurring during the cardiac cycles, from depolarization to repolarization of the cardiac muscle. This heartbeat has a peculiar appearance that comprises three different properties, including a P-wave representing the process of atrial depolarization, a QRS complex representing the process of ventricular depolarization, and a T-wave representing the process of ventricular repolarization [2]. The portions between these waves are called segments. Significant segments include the PR, ST, and TP segments, as illustrated in Fig 1.

Arrhythmias are a group of cardiac disorders characterized by irregular heartbeats. These include sinus bradycardia (SB), atrial tachycardia (AT), and irregular rhythms with missing or distorted wave segments and intervals, such as premature ventricular contraction (PVC). Of these, atrial fibrillation (AFIB) is the most prevalent and clinically important arrhythmia. It is characterized by an irregular, often rapid heartbeat and significantly raises the risk of cardiovascular complications, contributing to high morbidity and mortality [3]. The rising prevalence of AFIB is linked to aging populations and risk factors like hypertension and diabetes [4]. AFIB can cause serious complications, such as stroke, heart failure, and increased mortality[5]. The irregular heartbeat can lead to blood clots, which, if they travel to the brain, can cause strokes [6].

Past research [7–9] has focused on differentiating AFIB from sinus rhythm (SR). Kennedy *et al*. [7] suggested the application of Random Forest (RF) and K Nearest Neighbors (KNN) in the classification of AFIB and SR by applying the coefficient of sample entropy (CoSEn), the coefficient of variance (CV), root mean square of the successive differences (RMSSD), and median absolute deviation (MAD). Zhu *et al*. [8] proposed a novel technique based on maximum margin clustering, immune evolutionary algorithms, and wave and segment metrics for classifying ectopic heartbeats in the MIT-BIH database. Asgari *et al*.[9] suggested employing an SVM model to classify AFIB using the peak-to-average power ratio and log-energy entropy.

Recently developed deep learning methods can now match clinical cardiologists’ performance in ECG analysis and diagnosing arrhythmia diseases [10, 11]. However, obtaining high-quality, real-world ECG data for deep learning models is very challenging, mainly because of noise, variability in signal morphology, and artifacts obscuring crucial features. Furthermore, the imbalance in the class distribution of arrhythmia in a dataset increases the inherent complexity of learning from ECG signals since rare conditions do not have enough representation to train a robust model. These limitations have confined most deep-learning models to a small percentage of cardiac arrhythmia identification. Therefore, due to dataset structures and annotations, many research works focus primarily on heartbeat classification [12, 13].

These approaches often rely on minimal publicly available training datasets, making them challenging to deploy in real-world applications.

Integrating artificial intelligence into healthcare operations and services necessitates considering the inclusion of medical data from edge devices [14]. However, acquisition of such data is challenging due to its sensitivity and limited access [15]. Issues such as inadequate use, improper storage, data leaks, or exposure of personal information can compromise patient privacy. While data anonymization offers a potential solution, it is well-documented that removing identifiers such as a patient’s name or birth date often fails to ensure complete privacy [16].

Federated learning (FL) offers a promising solution by enabling collaborative model training on decentralized datasets without data sharing, thereby preserving privacy. In healthcare, it helps hospitals improve arrhythmia prediction models using high-resolution ECG signals without disclosing sensitive patient information. Unlike traditional methods, which require large datasets to be transmitted to centralized servers—demanding significant bandwidth—FL keeps data local and requires only minimal connectivity for model updates. Models trained via FL are inherently diverse, which enhances their generalization capabilities.

Multivariate signal processing techniques, specifically those dedicated to biomedical time-series data, are gaining attention because they can efficiently tackle complex inter-channel relationships in multi-channel signal data. Techniques such as Multivariate Empirical Mode Decomposition (MEMD), Joint Time-Frequency Analysis, and Multichannel Singular Spectrum Analysis have been cited as effective in isolating physiological information from high-dimensional ECG data [17, 18]. These techniques better capture inter-channel relationships and time-frequency attributes that are directly relevant to interpreting multi-lead ECGs. For example, adaptive decompositions and tensor models have been utilized to reveal underlying signal structures relevant to arrhythmia classification [19]. While analytically strong, these methods have several practical limitations. Most importantly, they rely on centralized access to datasets, which is typically infeasible in many healthcare settings due to patient confidentiality laws and institutional data silos. Second, these methods often require computationally intensive preprocessing, making them unsuitable for low-power edge devices used in real-time clinical monitoring [20]. Third, standard models based on these methods are not personalized; they use a single, universal classification model for all individuals, ignoring inter-subject variability in ECG measurements and demographic differences that significantly affect model generalization.

Some previous studies have attempted to address these challenges. For example, Kennedy *et al*. [7] used RR intervals along with multivariate statistical classifiers to detect atrial fibrillation, while Asgari *et al*. applied wavelet transforms combined with support vector machines (SVMs). Although informative, these approaches are limited by shallow learning architectures and a reduced ability to learn from heterogeneous environments.

As a result, deep learning architectures have been increasingly adopted for ECG analysis. CNN-based models, in particular, have demonstrated strong performance in recognizing spatial patterns [11]. However, CNNs are less effective at capturing the long-term temporal dependencies inherent in sequential physiological signals. Hybrid models that integrate CNNs with RNNs or attention mechanisms have shown improved performance, yet they still depend on centralized training and data availability, which limits their applicability in privacy-focused, real-world healthcare environments [21].

This study proposes a Fusion of Federated Learning with Personalized Models with Differential Privacy (FLPMDP) for arrhythmia classification using 12-lead ECGs. The combined approach of FL and differential privacy (DP) provides a revolutionary method for analyzing ECG data to detect arrhythmias. FL enables collaborative model training across several institutions without necessitating the exchange of raw data, thereby safeguarding patient privacy and complying with complex data protection regulations [22]. This capability has been significant in arrhythmia identification distributed ECG datasets by partitioning the number of clients based on the number of hospitals in scenarios 3 and 4 in the result and discussion section, improving diagnostic accuracy while protecting data[23]. While current multivariate methods exhibit excellent signal decomposition capabilities, they are incompatible with privacy preserving, real time, and decentralized learning systems, making them less useful in multi-center healthcare implementations. Conventional FL models, as efficient as they are, tend to overlook the clients’ heterogeneity and settle for suboptimal convergence in non-IID scenarios. The personalized local modeling in FLPMDP tackles this issue head-on by learning global knowledge and fitting it to clients’ unique data distributions. Furthermore, while incorporating differential privacy into FL typically comes at the cost of reduced performance, our model alleviates this issue by strategically applying Gaussian noise injection and L2 gradient clipping, thereby ensuring the optimal trade-off between privacy protection and model performance. Above all, the findings of our experiments—especially those under Scenario 4 illustrate that FLPMDP not only preserves stringent privacy preservation but also enjoys enhanced classification performance, thereby establishing its practical viability for deployment in real-world clinical environments. The empirical evaluation performed over four different experimental setups offers strong evidence of the advantage of the suggested FLPMDP model. In particular, the FLPMDP model attained a 98.71% holistic classification accuracy, complemented by 0.98 F1-score for sinus bradycardia (SB), 0.93 F1-score for atrial fibrillation (AFIB), 0.88 F1-score for supraventricular tachycardia (GSVT), and 0.89 F1-score for sinus rhythm (SR), clearly beating centralized and classical federated baselines. The results indicate the model’s ability to learn strong temporal and statistical patterns from 12-lead ECGs and highlight its ability to ensure high diagnostic performance in a privacy-conscious, decentralized setting. Moreover, the FLPMDP architecture was shown to be competitive in a variety of metrics such as specificity (up to 0.98), Kappa score 0.8971, and Matthews Correlation Coefficient 0.9042, thus testifying to its consistency and clinical reliability over all arrhythmia classes.

The subsequent sections of this paper are organized as follows. Sect 2 outlines the materials and methods employed in the proposed approach, Sect 3 presents the FLPMDP alogrithm to diagnose arrhythmia, Sect 4 describes the experimental setup, presenting the results and their interpretation as the empirical basis, Sect 5 outlines the limitations, and finally, Sect 6 concludes the paper, summarizing the key findings and suggesting directions for future research.

## 2 Proposed methodology

**Fig 2:**
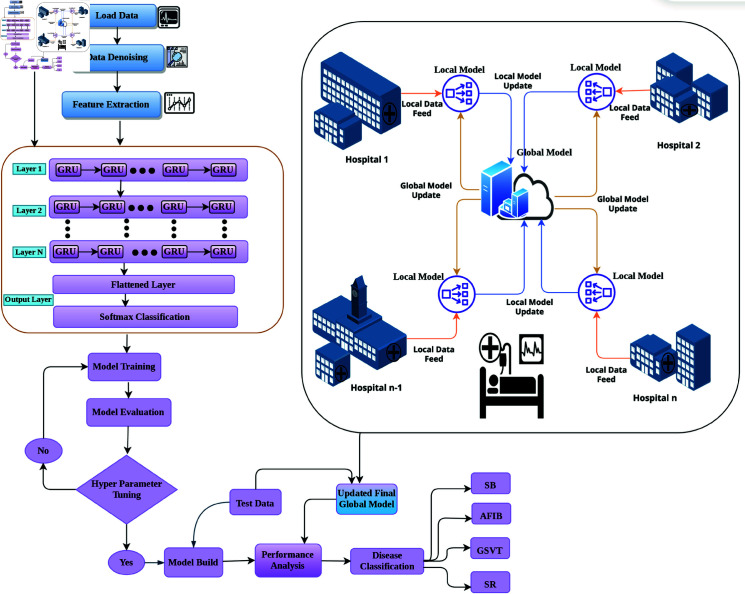
The proposed framework.

This section presents the proposed research methodology, including a description of the chosen dataset, its preprocessing, and the architectures of the proposed models. The proposed framework is shown in Fig 2. This research centers on a consortium of healthcare institutions that utilize low-powered medical devices and aim to train an artificial intelligence-driven module for arrhythmia classification while preserving the confidentiality of patient data. We suggest implementing a localized module within each organization to facilitate medical record collection, storage, and analysis. Each institution is assumed to possess high-definition ECG monitoring devices capable of accurately recording patient cardiac activity. There will be an export to an organization’s secure database of a 10-second 12-channel ECG recording from the device upon conducting monitoring organization . FL utilized in this research is a specialized machine learning approach that utilizes datasets distributed across multiple devices, ensuring data privacy and preventing leakage. It is a privacy-preserving, decentralized collaborative learning technique [24].

Healthcare professionals review ECG readings and write a diagnosis in a local database. A healthcare organization is assumed to have used a single and highly respected global cloud server. The global server allows the coordination of all actions performed by modules localized at each site. Medical records do not reside on the worldwide server. Only the core AI model and system parameters are preserved. A global server periodically organizes the global training sessions. It informs all the participating organizations, and devices within the organizations undergo local training. After completing pipeline processing at all local servers, it coordinates the local model training at the global server. Model weights are then sent back to the globalserver. The global server sends the final model to all organizations when all individual weights are summed. It continues until the distributed optimization has converged or several iterations have been reached. The following subsections summarize the problem formulation, data preparation, and the working principles of the Centralized Gated Recurrent Unit Model used in the proposed model.

### 2.1 Problem formulation

We assume that an ECG recording *s*_*i*_ consists of c∈[1,12] channels, each with a fixed sampling rate, resulting in *d* samples per channel. The recording can be represented as $s_{i}=\{x_{1}^{c},\ldots ,x_{d}^{c}\}$ for each channel *c*, where $x_{j}^{c}$ denotes the *j*-th sample of channel *c*, with j∈[1,d]. The multiclass ECG arrhythmia classification task addressed in this study takes as input a dataset of *n* ECG recordings, each consisting of 12 channels (denoted as $S=\{s_{1},\ldots ,s_{n}\}$). The output is a sequence of labels $L=[l_{1},\ldots ,l_{n}]$, where each label *l*_*i*_ belongs to the set of arrhythmia classes under consideration.

### 2.2 Data preparation

The dataset in [25] is utilized in this study, consisting of 10,646 ECG recordings from 5,956 men and 4,690 women, 11 heart rhythms, and 56 cardiovascular diseases diagnosed by doctors. The data includes key ECG measurements, such as patient age, gender ventricular rate, atrial rate, QRS durations, QT interval, QT corrected, Raxis, Taxis, QRS count, Qonset, Qoffset, and Toffset, providing comprehensive insights into the patient’s cardiovascular health.

There were four stages in the data acquisition process. First, each subject recorded a 10-second 12-lead ECG test in the GE MUSE system. Then, a second-stage physician labeled the rhythms and conditions, with another validating them. In case of disagreement, a senior physician made a final call. Labels were rhythms and conditions, including PVCs, RBBBs, LBBBs, and APBs. Each condition was applied to the sample rather than the individual beats within 10 seconds. Thirdly, this study found that ECG data contamination included power line interference, noise from electrode contact, motion artifacts, muscle contraction, baseline wandering, and random noise, which can impede analysis. A sequential noise reduction approach was used. First, a Butterworth low-pass filter eliminated frequencies above 50 Hz, matching the normal ECG range (0.5 Hz to 50 Hz). LOESS [26, 27] smoothing reduced the baseline wandering. The technique of Non-Local Means (NLM) [28] removed the leftover noise for smoother analysis—finally, the data was stored in CSV file format.

For classification purposes, some rare rhythms with fewer than 10 samples were merged into broader categories, as suggested by cardiologists. The 11 rhythms were grouped into four categories: SB, AFIB, GSVT (Supraventricular Tachycardia and other related rhythms), and SR. Hierarchical grouping increases the classification of unusual cases while retaining clinical applicability.

### 2.3 Centralized gated recurrent unit model

A gated Recurrent Unit (GRU) is a Recurrent Neural Network (RNN) type for sequential data processing and is highly suited for classifying arrhythmia from 12-lead ECG signals. GRU solves the problem of vanishing gradients, a common problem in traditional RNNs, due to gating mechanisms that efficiently capture the long-term dependency [29, 30]. The GRU model takes in ECG readings from 12 leads. It captures the features by the gates and trains on labeled ECG data to identify arrhythmia patterns. It updates and resets information in the gates at every step, learning normal and abnormal heart rhythms. The GRU operates by processing the input sequence and updating its hidden state at each time step [31]. It uses two key gates, the update gate and the reset gate, which control how much of the previous state should be retained and how much of the new input should be incorporated [32]. The update gate decides how much of the previous memory should be maintained at each time step *t* and is defined as

$$z_{t}=\sigma (W_{z}x_{t}+U_{z}h_{t-1}+b_{z})$$ (1)

where *x*_*t*_ is the input at time step *t*, *h*_*t*−1_ is the previous hidden state, *W*_*z*_ and *U*_*z*_ are weight matrices, *b*_*z*_ is the bias term, and σ is the sigmoid activation function that outputs values between 0 and 1. The reset gate determines how much of the previous hidden state should be forgotten and is defined as

$$r_{t}=\sigma (W_{r}x_{t}+U_{r}h_{t-1}+b_{r}).$$ (2)

The reset gate also uses the sigmoid function to control the information retained from the previous state.

The candidate hidden state is computed by combining the current input and the influence of the reset gate on the previous hidden state and is defined as

$${\tilde{h}}_{t}=\tanh(W_{h}x_{t}+U_{h}(r_{t}⊙h_{t-1})+b_{h})$$ (3)

where tanh represents the hyperbolic tangent activation function, and ⊙ denotes element-wise multiplication.

The final hidden state *h*_*t*_ at time step *t* is calculated by a weighted combination of the previous hidden state and the candidate hidden state, controlled by the update gate

$$h_{t}=(1-z_{t})⊙h_{t-1}+z_{t}⊙{\tilde{h}}_{t}$$ (4)

This equation shows how the update gate (*z*_*t*_) blends the previous hidden state and the candidate hidden state. When *z*_*t*_ is close to 1, more of the previous hidden state is retained, and when *z*_*t*_ is close to 0, more reliance is placed on the candidate’s hidden state.

In our approach, GRU was selected over LSTM as GRU has proven to be computationally efficient but still performs well on time-series data, like ECG signals, which is essential for arrhythmia diagnosis [20]. Compared to LSTM, the GRU is simpler in architecture and has fewer parameters; it is memory-efficient and faster to train without sacrificing the model’s performance. This feature is handy in FL scenarios, where clients would have scarce resources and communication constraints. Recent research explains that GRU models have proven to be robust for time-series classification tasks with lower memory usage and faster convergence than LSTM models [20]. Also, while LSTM is widely used in time-series tasks, we discovered that GRU performed just as well in our specific setup but with the advantage of lower computational overhead. GRU is thus a prime candidate for time-sensitive applications like real-time arrhythmia monitoring.

## 3 Federated Learning with Personalized Models with Differential Privacy (FLPMDP)

The proposed hybrid model uses FLPMDP to diagnose arrhythmia using 12-lead ECG signals with excellent accuracy and data privacy. Edge devices and hospitals train individualized models on local ECG data in FLPMDP, which are aggregated into a global model. Raw data stays at the client’s premises, protecting privacy. DP protects patient data by adding Gaussian noise to gradients during training. A multiplier and L2 norm clipping reduces noise, boosting privacy and model correctness across rounds[33] In FLPMDP, noise is added to gradients during model training to hide the effect of individual input points. For client *i*, let *g*_*i*_ represent the loss function gradient about model parameters, then DP is enforced by adding Gaussian noise $𝒩(0,\sigma^{2})$ to each gradient as

$${\tilde{g}}_{i}=g_{i}+𝒩(0,\sigma^{2}).$$ (5)

The standard deviation of Gaussian noise, σ, is governed by the privacy budget ϵ, balancing privacy with model accuracy. We calculate the noise multiplier σ as:

$$\sigma =\frac{C\cdot \text{Sensitivity}}{\epsilon }.$$ (6)

In the Gaussian noise mechanism, *C* is a constant, and sensitivity is the greatest change in gradient owing to a single data point inclusion or exclusion. Each FL client trains a local dataset-specific model. After training, the central server aggregates local model updates to generate the global model. We aggregate using Federated Average Fed_avg, given by

$$\theta_{\text{new}}=\sum_{i=1}^{N}\frac{\left|D_{i}\right|}{\left|D\right|}\cdot \theta_{i}$$ (7)

where $\theta_{\text{new}}$ represents the aggregated global model parameters, $\theta_{i}$ represents client parameters, |*D*_*i*_| represents client’s dataset samples, and |D| represents all client samples.

DP in FL requires clipping gradients to a set L2 norm to avoid outliers from dominating gradient updates. The cutting procedure is given by

$$g_{i}^{\prime }=\min\left(1,\frac{\text{Clip}(g_{i})}{C}\right).$$ (8)

where $\text{Clip}(g_{i})$ is the client gradient, and *C* is the clipping threshold. This prevents gradients from having huge values that might compromise privacy.

Equation 5 adds Gaussian noise to gradients after clipping before aggregating them at the central server. The global model is updated using noisy gradients, preventing global model changes from leaking crucial data point information.

The loss function used for training is typically a cross-entropy loss for classification problems like arrhythmia detection. The optimization algorithm for updating the model parameters is stochastic gradient descent (SGD) or Adam, modified to account for DP. Mathematically, the objective function in a FL setting is given by

$$ℒ(\theta )=\frac{1}{N}\sum_{i=1}^{N}{ℒ}_{i}(\theta )$$ (9)

where ${ℒ}_{i}(\theta )$ is the local loss function for client *i*, and *N* is the number of clients.

After the local training, all the clients transfer their updated model parameters to a centralized server, and then Fed_avg aggregates those parameters according to the sample size. This creates a global model incorporating knowledge from all clients without accessing raw data. This global model then gets fine-tuned locally by each client to get further customized to specific local patterns. Finally, the performance of both global and personalized models is measured over a standard test set based on their capability to diagnose arrhythmia effectively yet privately. Algorithm 1 shows this FLPMDP algorithm.

## 4 Results and discussion

The following subsections discuss the experimental setup, analysis across different scenarios, and benchmarking with existing work from the literature.

### 4.1 Experimental setup

To ensure reproducibility and transparency about the experimental environment, all training and evaluation processes related to the model were conducted using Python 3.10.8 as the primary execution environment, and PyTorch and Keras (version 2.6.0) were the primary deep learning libraries. The experiments were performed on an HP ZBook Studio G3 workstation, which was outfitted with an Intel Core i7 processor belonging to the 6th Generation, 16 GB of Random Access Memory (RAM), and an NVIDIA GPU with 4 GB of local memory. A collection of supporting Python libraries was utilized to ease the execution of different computational processes: NumPy version 1.19.5 for numerical methods, Scikit-Image version 0.18.1 for image processing processes, and Scikit-Learn for executing machine learning processes. The Pandas, Matplotlib, and Seaborn libraries executed data manipulation and visualization procedures because of FL, which involves decentralized model training on multiple clients; measuring execution time as a major performance indicator is significant. Execution time, measured in seconds, was determined as the duration between the initialization of local model training and the completion of results aggregation. The hardware specifications utilized in this research are presented in Table 1.

**Table 1:** Hardware and software specifications used in the experimental setup.

Component	Specification
Workstation Model	HP ZBook Studio G3
Processor	Intel(R) Core(TM) i7-6700HQ CPU @ 2.60GHz (8 CPUs), ~2.6GHz
Memory (RAM)	16 GB
GPU	NVIDIA Quadro M1000M, 4 GB VRAM
Operating System	Windows 10 Pro 64-bit (Build 19045)
Python Version	3.10.8
Deep Learning Libraries	PyTorch, Keras 2.6.0
Supporting Libraries	NumPy 1.19.5, Scikit-Image 0.18.1, Scikit-Learn
Visualization Libraries	Seaborn, Matplotlib
Data Handling Library	Pandas

### 4.2 Analysis across different scenarios

This section outlines the results and analysis carried out using four different scenarios. A complete categorization report for the four scenarios is provided, and sensitivity and specificity are discussed in the subsequent sections. The dataset is divided into 75% for training, 10% for validation, and 15% for testing. We used the raw ECG data in the first scenario to implement the GRU model. We integrated 13 features with the raw ECG data for arrhythmic disease classification in the second scenario. The third scenario used the same parameters and scheme as the second scenario, adding weighted Fed_avg. Finally, in the fourth scenario, we applied the FLPMDP methodology, which also used the same parameters and feature fusion scheme of the second scenario. The details of the parameters and schemes for all scenarios are discussed below.

**Algorithm 1** Hybrid Personalized Federated Learning with Differential Privacy (DP)

**Require:** Client datasets $\{D_{i}{\}}_{i=1}^{n}$, global model

  𝒢, GRU model parameters, learning rate η, noise multiplier

  σ, gradient clipping norm *C*, number of global epochs *E*_*g*_,

  number of local epochs *E*_*l*_

**Ensure:** Trained global model 𝒢 and personalized models $\{{𝒞}_{i}{\}}_{i=1}^{n}$

1: **Initialize:** Global model 𝒢 and client models $\{{𝒞}_{i}{\}}_{i=1}^{n}$

2: Initialize noise multiplier σ and clipping norm *C*

3: **for** *t* = 1 **to** *E*_*g*_ **do**

4:   {Global rounds} 5:   Adjust σ and *C* dynamically

6:   Set global parameters ${𝒲}_{t}=0$

7:   **for** each client i∈{1,…,n} **do**

8:    {Client-side training}

9:    Load 𝒢 weights into ${𝒞}_{i}$

10:    **for** *e* = 1 **to** *E*_*l*_ **do**

11:     **for** each batch in *D*_*i*_ **do**

12:      Compute loss and gradients

13:      Clip gradients using norm *C*

14:      Add Gaussian noise with multiplier σ

15:      Update ${𝒞}_{i}$ using noisy gradients

16:     **end for**

17:    **end for**

18:    Store client weights *W*_*i*_ and local data size *N*_*i*_

19:   **end for**

20:   Compute weight factors $w_{i}=\frac{N_{i}}{\sum_{j}N_{j}}$

21:   Aggregate updates: $𝒢=\sum_{i}w_{i}W_{i}$

22:   Evaluate 𝒢 on global test set

23:   **for** each client *i* **do**

24:    {Personalization phase}

25:    Fine-tune ${𝒞}_{i}$ using 𝒢

26:    Evaluate personalized ${𝒞}_{i}$

27:   **end for**

28: **end for**

29: Save final global model 𝒢

30: **Model Evaluation:** Compute Accuracy, Precision, Recall, and F1-score

31: **return** Final performance metrics

#### 4.2.1 Scenario 1.

**Fig 3:**
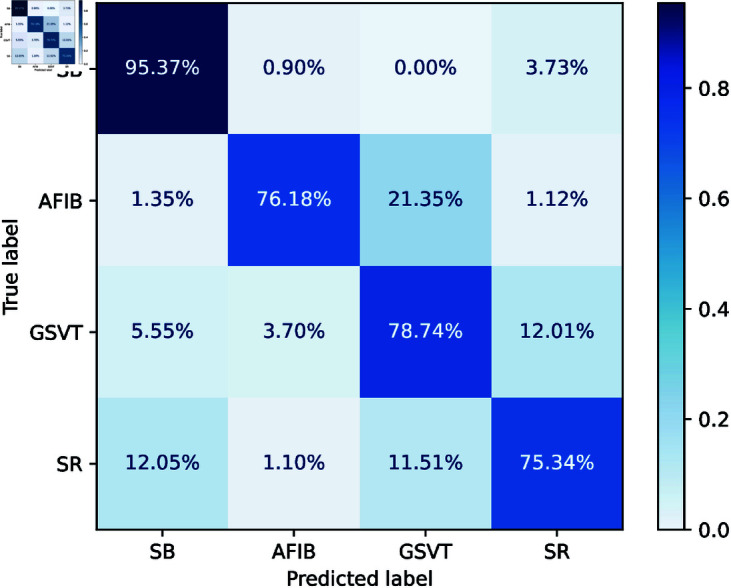
Confusion matrix of GRU without Feature Fusion Model.

The first scenario utilized a 12-lead ECG dataset to develop a GRU model for arrhythmia classification without incorporating feature fusion. The GRU-based network was optimized for arrhythmia classification by a systematic hyperparameter tuning process exploring hidden layer sizes [32,48,64,128], learning rates [0.01,0.001,0.0001], and GRU layers [3,4,5,6,7]. This resulted in the optimal configuration of hidden layer size = 64, number of GRU layers = 5, and learning rate = 0.001, which showed the best trade-off between accuracy and generalization. The input size was set to 12, representing the 12-lead ECG features, while the output size corresponded to 4 arrhythmia classes (SB, GSVT, AFIB, SR). The model used cross-entropy loss with label smoothing of 0.2 to avoid overconfidence and Adam optimizer with weight decay 1 × 10^−5^ for adaptive learning and regularization. This setup ensures robust performance, well capturing temporal dependencies without suffering from overfitting, and improved accuracy and generalization in arrhythmia classification. The confusion matrix is given in Fig 3 that shows how efficiently GRU identifies SB, AFIB, GSVT, and SR arrhythmia. SB has the best classification accuracy, recognizing 95.37% of SB instances with certain misclassified cases. AFIB performs similarly with 76.18% sensitivity and 21.35% GSVT misclassifications. GSVT has 78.74% accuracy and 12.01% SR ambiguity. SR classification is 75.34% correct.

The classification report in Table 2 further corroborates these findings. SB has the highest recall of 0.95 and F1-score of 0.93 for prediction. AFIB achieves a precision of 0.92, but a poor recall of 0.76 drops AFIB’s F1-score to 0.83, showing difficulties in identifying all positive events. GSVT and SR show the lowest precession, F1 score, and accuracy records.

**Table 2:** Performance comparison of GRU model with and without Feature Fusion.

Class Label	Without Fusion (P / S / Sp / F1)	With Fusion (P / S / Sp / F1)
SB	0.90 / 0.95 / 0.94 / 0.93	0.98 / 0.98 / 0.99 / 0.98
AFIB	0.92 / 0.76 / 0.98 / 0.83	0.91 / 0.95 / 0.98 / 0.93
GSVT	0.76 / 0.79 / 0.89 / 0.77	0.95 / 0.86 / 0.98 / 0.90
SR	0.74 / 0.75 / 0.94 / 0.74	0.85 / 0.94 / 0.96 / 0.89

#### 4.2.2 Scenario 2.

In this scenario, arrhythmia classification is conducted by a hybrid GRU-based model that simultaneously processes temporal ECG signals and fused features. This work optimized the model parameters by employing the hyperparameter tuning approach addressed in Scenario 1 and, further, by blending 13 feature-derived characteristics simultaneously. Eventually, after the hyperparameter tuning, the model’s final setting was a hidden size of 48 with 5 GRU layers, which let the model process 12-lead ECG data directly within the GRU architecture. The Adam optimizer has a learning rate = 0.001, weight decay 1 × 10^−5^, and CrossEntropyLoss criteria with label smoothing 0.2 to optimize and train the model. The model extracted sequential and feature-based information by incorporating parallel feature fusion, enhancing discrimination of arrhythmia classification. The technique demonstrated that raw signal processing with complementary features could balance computing efficiency and classification accuracy for challenging arrhythmia detection applications.

**Fig 4:**
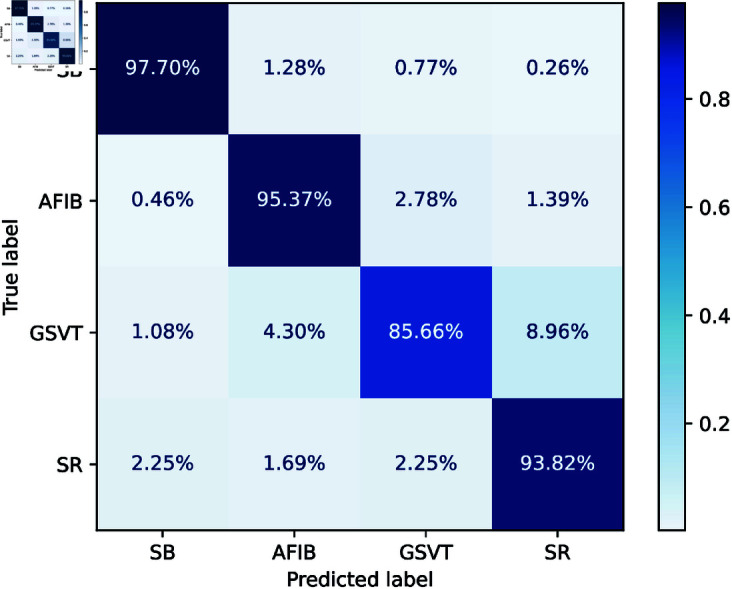
Confusion matrix of GRU model with Features Fusion.

Feature fusion improves classification accuracy, as per the confusion matrix given in Fig 4. SB has 97.7% accuracy with few misclassifications. AFIB improves highly, as 95.37% of instances are identified with minimum confusion. GSVT reduces SR misclassification to 8.96% with 85.66% accuracy. SR categorization significantly improves with 93.82% accuracy and low-class confusion. These findings show that feature fusion improves GRU model performance by boosting class separability and reducing misclassification rates across all arrhythmia classes.

SB is very accurate with near-perfect precision, recall, and F1-score 0.98, given in Table 2. High sensitivity (0.95 and 0.94) helps AFIB and SR achieve F1-scores of 0.93 and 0.89. GSVT’s F1-score is 0.90 with a balanced accuracy of 0.95 and recall of 0.86.

#### 4.2.3 Scenario 3.

In scenario 3, we investigated an extended GRU model with feature fusion for arrhythmia classification, integrating FL through a weighted averaging strategy. A weighted technique aggregates model weights across remote nodes via Fed_avg. This solution uses FL to protect patient privacy in healthcare applications while retaining good classification performance across datasets. Our methodology overcomes data-sharing restrictions and decreases data storage reliance by decentralizing training. Adding FL makes arrhythmia detection scalable and privacy-preserving, making it more applicable in clinical settings.

**Fig 5:**
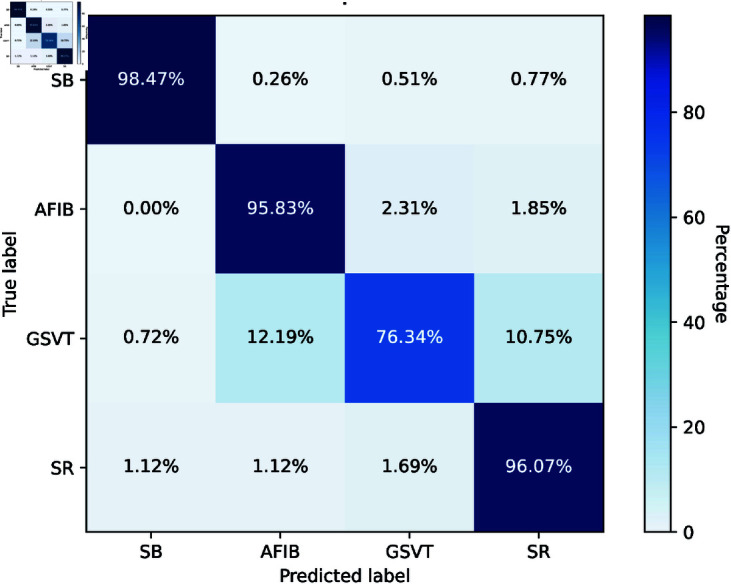
Confusion matrix of Fed_avg Model with Features Fusion.

The confusion matrix in Fig 5 demonstrates that the federated weighted average model excelled in diagnosing four diseases: SB, AFIB, GSVT, and SR. It achieves excellent accuracy for SB 98.47% and SR 96.07%, with minor misclassification. Nonetheless, there was a considerable misunderstanding between GSVT and AFIB, shown by the 12.19% misclassification of GSVT as AFIB.

Table 3 gives a classification report with feature fusion that exhibits robust performance in a four-class illness classification challenge. SB attained the maximum precision of 0.99, recall of 0.98, and F1-score of 0.99, signifying exceptional accuracy. AFIB and SR exhibited strong performance with F1-scores of 0.90 and 0.89, respectively, underscoring their excellent sensitivity and specificity. GSVT had a little reduced recall of 0.76 while maintaining a commendable F1-score of 0.85. The specificity remained continuously elevated across all categories, highlighting the model’s proficiency in reliably identifying real negatives.

**Table 3:** Performance Comparison of Fed_avg and FLPMDP Models.

Class Label	Weighted Fed_avg (P / S / Sp / F1)	FLPMDP (P / S / Sp / F1)
SB	0.99 / 0.98 / 0.99 / 0.99	0.97 / 0.99 / 0.98 / 0.98
AFIB	0.85 / 0.96 / 0.96 / 0.90	0.89 / 0.95 / 0.97 / 0.92
GSVT	0.96 / 0.76 / 0.99 / 0.85	0.91 / 0.85 / 0.96 / 0.88
SR	0.82 / 0.96 / 0.96 / 0.89	0.89 / 0.89 / 0.98 / 0.89

#### 4.2.4 Scenario 4.

In this last scenario, the proposed scheme FLPMDP tackles the simultaneous problem of safeguarding data privacy while facilitating collaborative learning across distant devices. In healthcare, where sensitive patient information must be protected , FLPMDP guarantees strong model training without disclosing individual records. This approach is proposed to improve privacy-preserving features while ensuring high classification accuracy, especially for multi-class illness detection in decentralized medical systems.

**Fig 6:**
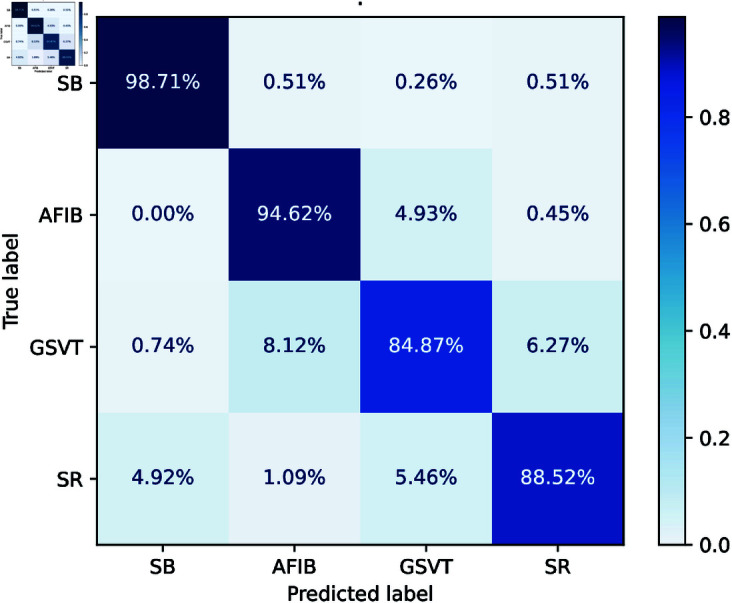
Confusion matrix of FLPMDP model with Features Fusion.

The confusion matrix given in Fig 6 indicates that the FL model with DP successfully classified four illness categories. SB had a high accuracy of 98.71%, and AFIB demonstrated 94.62% accuracy, whereas significant misclassification occurred in SR, with 11.47% incorrectly identified, including 4.92% categorized as SB. The GSVT classification showed enhancement, achieving an accuracy of 84.87% and a decreased confusion rate of 8.12% with AFIB.

The FLPMDP results are presented in Table 3. SB achieved remarkable outcomes with a sensitivity of 0.99, specificity of 0.98, and an F1-score of 0.97, underscoring precise predictions. AFIB had a sensitivity of 0.95, a specificity of 0.97, and an F1-score of 0.93. GSVT exhibited a somewhat reduced sensitivity of 0.85 while maintaining a reasonable specificity of 0.96 and a robust F1-score of 0.87. SR exhibited a balanced performance, achieving a sensitivity of 0.89, a specificity of 0.98, and an F1-score of 0.90.

**Fig 7:**
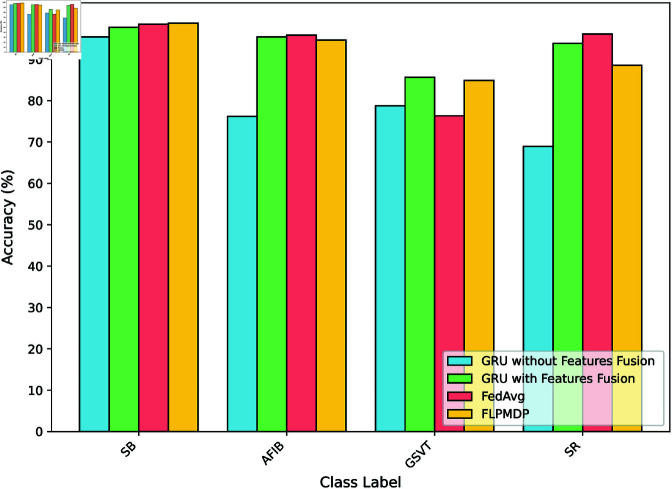
Model accuracy across different classes.

The accuracy comparison across different arrhythmia classes for each model is illustrated in Fig 7. The x-axis represents the disease classes, while the y-axis indicates their corresponding accuracy percentages.

The GRU model without feature fusion, shown by sky blue bars, has moderate performance, with accuracy ranging from 68.92% to 95.37%. Conversely, the GRU model with feature fusion (light green bars) attains superior accuracy, ranging from 85.66% to 97.70%. The Fed_avg model (light coral bars) demonstrates robust performance, with accuracies ranging from 76.34% to 98.47%. The FLPMDP model (salmon bars) outperforms all other models, constantly attaining high accuracy rates, with a maximum of 98.71%.

### 4.3 Comparison with existing approaches and computational overhead

Table 4 provides a comprehensive comparison to facilitate quantitative and clear evaluation of our proposed method against existing models. The table shows that FLPMDP outperforms well-known models, including CNN, ResNet, and Fed_avg-based models, in terms of accuracy, precision, recall, F1-score, and specificity. Specifically, FLPMDP boasts an impressive 93.71% accuracy, surpassing Weighted ResNet Fed_avg at 68.4% and Fed Neural Networks Fed_avg with a projected accuracy of approximately 68.1%. Further, FLPMDP achieves a precision of 0.92, is greater than benchmark models such as Self-supervised learning 0.726 and Fed_avg(GAN) 0.86, indicating its strong capability for making accurate optimistic predictions. FLPMDP’s recall, or sensitivity, is 0.92, which is equal to or greater than other methods, such as TERMA 0.890 and CNN-RNN-attention 0.9179, demonstrating FLPMDP’s strong capacity for identifying positive arrhythmia cases.

**Table 4:** Performance comparison with existing models.

Method	Accuracy	Precision	Recall / Sensitivity	F1-Score	Specificity
Weighted ResNet Fed_avg [34]	68.4%	-	0.758	-	0.969
TERMA [35]	87.0%	0.900	0.890	89.5%	-
CNN [36]	92.5%	-	~0.90	-	-
Fed Neural Networks Fed_avg [37]	~68.1%	-	~0.72	0.71–0.78	-
OMOP-CDM [38]	88.17%	0.870	0.890	0.890	-
CNN-RNN-attention [39]	84.65%	0.8494	0.9179	0.8819	0.7279
Fed avg (GAN) [40]	89.0%	0.86	-	~0.85	-
Self-supervised learning [41]	90.0%	0.726	0.872	0.720	0.959
**Proposed Methodology**					
**GRU_Fusion**	**93.14%**	**0.92**	**0.93**	**0.93**	**0.98**
**Weighted Fed_avg**	**92.19%**	**0.91**	**0.92**	**0.91**	**0.98**
**FLPMDP**	**93.71%**	**0.92**	**0.92**	**0.92**	**0.98**

FLPMDP F1-score 0.92 also reflects an optimal trade-off between precision and recall, which is highly desirable in medical diagnoses. Compared to alternatives such as Self-supervised learning and CNN, which yield lower F1-scores of 0.720 and approximately 0.90, respectively, FLPMDP has a better trade-off between the two critical metrics. FLPMDP also has a specificity of 0.98, which is better than other models, such as CNN-RNN-attention 0.7279, reflecting its strong capability to correctly classify negative samples in the data. Apart from emphasizing the superiority of FLPMDP, it is essential to point out that other methods, such as Weighted ResNet Fed_avg and Federated Neural Networks Fed_avg, are significantly poorer, with accuracy rates of 68.4% , and 68.1%, respectively. Furthermore, these methods exhibit relatively lower recall and F1 scores, making them less suitable for clinical use, where both high accuracy and sensitivity are crucial.

In our evaluation, Centralized Learning (CL) recorded the highest Kappa score of 0.9044, indicating an outstanding concordance between predicted results and actual labels as given in Table 5. FLPMDP recorded a closely related Kappa score of 0.8971, indicating high performance with the privacy feature intact through applying differential privacy methods. While Weighted Fed_avg recorded a lower Kappa score of 0.8910, the variation is minimal and does not significantly impact the model’s overall effectiveness. Similarly, in terms of the Matthews Correlation Coefficient (MCC), a broadly regarded measure as being among the most informative metrics used to quantify models in instances of imbalanced datasets, CL recorded the highest score of 0.9075, with FLPMDP, with DP recording a close second at 0.9042, and Weighted Fed_avg at 0.8932. The findings indicate that FLPMDP with DP provides performance comparable to that of CL while, at the same time, providing data privacy, a critical consideration in healthcare environments where patient information confidentiality is a significant consideration.

**Table 5:** Computational overhead comparison between centralized learning and FL-based approaches.

Metric	Centralized Learning (Proposed)	Weighted Fed_avg (Proposed)	FLPMDP (Proposed)
Kappa Score	0.9044	0.8910	0.8971
MCC Score	0.9075	0.8932	0.9042
Training Time / Epoch	324.06 s	~2439.39 s (Global Round)	~2669.59 s (Global Round)
Testing Time / Epoch	20.8 s	25.8 s	27.8 s
Peak GPU Memory Usage	1379.60 MB	1853.27 MB	1943.27 MB
Approx. CPU RAM Usage	208.32 MB	872.86 MB	882.86 MB
Comm. Cost (per client/round)	None	~0.52 MB	~0.54 MB

The training duration per epoch within CL is significantly efficient at 324.06 seconds since all computing occurs within one centralized node as given in Table 5. In contrast, in FL based methodologies, the training time is considerable due to the requirement to complete various global rounds with communication between clients and the central server. The training time for the Weighted Fed_avg would be approximately 2439.39 seconds. In comparison, FLPMDP has a training time marginally higher at 2669.59 seconds, added to the supplementary privacy-providing mechanisms. Each epoch’s testing time is slightly higher for FL-based methods than for Centralized Learning. In particular, CL takes 20.8 s for testing, Weighted Fed_avg takes 25.8 s, and FLPMDP takes 27.8 s. This results from multiple clients’ processing overhead and the extra privacy measures utilized in the FLPMDP.

GPU memory consumption is a significant concern when training massive models as shown in Table 5. CL takes 1379.60 MB, while FL-based methods consume more memory to store the local models on each client and handle the global model updates. Weighted Fed_avg, in particular, takes 1853.27 MB, and FLPMDP takes 1943.27 MB because of the additional privacy-preserving computations.The CPU RAM usage is relatively low in CLat 208.32 MB. However, in FL-based approaches, CPU RAM usage is significantly high since additional local models must be saved, and the aggregation operation must be performed across clients. In Weighted Fed_avg, the usage is 872.86 MB, and in FLPMDP, the usage is 882.86 MB. One of the significant overheads in FL is communication cost, which is absent in Centralized Learning. For Weighted Fed_avg, the communication cost per client per round is ~0.52 MB, and for FLPMDP, it is ~0.54 MB. Though these are not very high costs, they play an essential role in FL as the number of clients and model size increases.

Although FLPMDP introduces extra computational overhead, these techniques are essential to privacy protection for healthcare applications, particularly for diagnosing arrhythmia. The ensuing training time, memory requirement, and communication overhead are all necessary compromises to safeguard the security and confidentiality of invaluable patient data throughout the learning process. FL ensures that the data remains decentralized on local clients, reducing the risk of privacy breaches, which is critical in healthcare domains. Hence, despite the overhead, FL is a very valuable method of preserving data privacy while maintaining strong classification performance, as confirmed by the competitive Kappa and MCC values we achieved through our experiments.

## 5 Limitations

Although our proposed PFL-DP framework proposes improved privacy-preserving personalized learning for arrhythmia diagnosis, it does not necessarily include contextual information such as patients’ activity levels, circadian rhythms, environmental conditions, or comorbidities that can strongly impact ECG signal interpretation. Not including such information could restrict the model’s generalizability and diagnostic performance within real-world clinical settings. Contextual data inclusion has been described in previous work as promoting improved diagnostic accuracy and model robustness of artificial intelligence [42]. For example, Zhang *et al*. [43] introduced a context-aware federated learning system based on wearable devices, Chen *et al*. [44] emphasized improved ECG-based diagnostic outcomes using patient-specific streams of contextual information, and Gupta *et al*. [45] implemented a multi-institutional study that yielded superior arrhythmia detection while employing contextual modeling. Future research will aim to develop hybrid frameworks that bring together context-aware techniques with PFL-DP to develop more clinically valid and reliable diagnostic systems.

## 6 Conclusion

This research presented a Hybrid FL with Personalized Models with Differential Privacy (FLPMDP) for identifying arrhythmia utilizing 12-lead ECG readings. This method guarantees data privacy via decentralized training and noise infusion while improving model generalization across diverse datasets. Experimental findings demonstrate increased accuracy, recall, and F1 scores across various arrhythmia classifications. GRU-based temporal analysis and feature fusion enhanced the model’s discriminative capability. Despite strong performance and scalability, class imbalance and communication costs need adjustment for broader clinical use. Future analyses will focus on improving the framework’s handling of imbalanced datasets and enhancing communication efficiency in FL. Additionally, incorporating real-time detection and explainable AI will further support clinical decision-making. In addition, we also intend to explore the application of Transformer-based models in future research, especially that of Personalized FL (PFL) with differential privacy. To investigate whether these models can provide additional benefits in performance and scalability for ECG-based arrhythmia diagnosis.
